# Like a bridge over troubled water: keeping the myeloma down en route to CAR-T

**DOI:** 10.1038/s41408-024-01049-z

**Published:** 2024-04-12

**Authors:** Joshua Richter

**Affiliations:** Tisch Cancer Institute: Icahn School of Medicine at Mount Sinai, New York, NY USA

**Keywords:** Cancer immunotherapy, Myeloma

Chimeric antigen receptor T-cell (CAR-T) therapy has become a key option for patients with relapsed and refractory Multiple Myeloma (RRMM). Due to a variety of logistical issues, patients may warrant “bridging” therapy between T-cell leukapheresis and lymphodepleting chemotherapy. This bridge may serve multiple roles: (1) Provide disease control during the manufacturing process (and avoid sequelae of progressive myeloma); (2) Potentially reduce CAR-T-related toxicities, which may correlate with disease burden; (3) potentially improve remission durability as a result [1]. Afrough et al. delineate the impact of bridging therapy in a real-world dataset from the U.S. Myeloma Immunotherapy Consortium [2].

On March 26, 2021, the Food and Drug Administration (FDA) approved idecabtagene vicleucel (Ide-cel) as the first CAR-T therapy for RRMM (https://www.fda.gov/drugs/resources-information-approved-drugs/fda-approves-idecabtagene-vicleucel-multiple-myeloma). Subsequently, on February 28, 2022 the FDA approved ciltacabtagene autoleucel (Cilta-cel) as an additional CAR-T for RRMM (https://www.fda.gov/drugs/resources-information-approved-drugs/fda-approves-ciltacabtagene-autoleucel-relapsed-or-refractory-multiple-myeloma). Despite the evolving data showing unprecedented response rates, in both early and heavily relapsed MM, there are a number of “logistical hurdles” that must be taken into account when considering these options for patients. Apheresis/production availability is the first impediment that must be overcome as there is, at this time, a limited number of slots for collection and manufacturing. Once the apheresis date is confirmed the general practice is to avoid chemotherapy for 2–3 weeks prior to apheresis in efforts to avoid altering T-cell health/number. Once the apheresis is complete the T-cell manufacturing and subsequent quality control protocols may require 3–5 weeks [3] (and in some cases up to 8 weeks); notwithstanding ongoing efforts to shorten this timeframe. To this end, RRMM patients would have, in some cases, a 2-month gap to overcome between confirmation of progressive disease and CAR-T infusion. As a first pass, to avoid sequelae of progressive disease, bridging therapy (BT) helps to ensure a higher chance that the patient does not have excessive morbidity/mortality during the vein-to-vein time. Although there may be highly restricted options for BT in the context of clinical trials; commercial BT is at the discretion of the treating physician and may vary wildly due to factors such as disease burden, kinetic/rate of progression, end-organ dysfunction, therapy availability, prior therapy tolerability and response, patient preference, efficacy/toxicity profile, presence of symptomatic plasmacytoma, etc.

Options for BT run the gamut from observation alone (for patients with low burden/well-controlled disease) all the way up to aggressive chemotherapy. Radiation for focal disease can be used as a bridging approach for RRMM [4], however, the corollary experience in lymphoma is a bit more flushed out [5, 6]. This dataset from the U.S. Myeloma Immunotherapy consortium, with 214 patients from 11 sites, represents one of the largest of a kind to date. Several conclusions arise from their work. Put simply…..less is more!! Patients not receiving/requiring BT had a significantly longer PFS (11.48 vs. 6.68 months), longer OS (not reached vs. 13.85 months), shorter hospital stay (10 vs. 8 days), and a non-significant trend towards lower rates of immune effector-cell neurotoxicity syndrome (ICANS).

Despite the best-laid plans of mice and men; patients may still require BT. In this scenario, the data is evaluated in three groups: No alkylators, weekly cyclophosphamide (Cy), and intensified/infusional-Cy. Notwithstanding that one of the major determinants of choice of BT is disease burden and growth kinetic; a number of interesting results arise. Although alkylator-based BT resulted in worse PFS and OS, when this was separated into weekly vs. intensified/infusional Cy the data showed that the weekly application was not statistically different from the non-alkylator group. Ultimately it was the more aggressive alkylator group that led to both worse PFS (4.6 vs. 12 months) as well as OS (10 vs. NR months) compared with the other BT approaches. This data corroborates findings from a smaller previous study showing overall worse outcomes from patients receiving/requiring more intensive alkylator-based BT [7].

In large B-cell lymphoma, it has been shown that patients requiring systemic BT will have inferior outcomes [8]. In RRMM higher disease burden (including extramedullary disease) as well as serologic markers of increased inflammation (ferritin, CRP, IL-6) are predictive for both increased acute toxicity as well as diminished clinical outcomes [9–11]. Although a personalized approach is still warranted, the overarching principle of disease control prior to CAR-T infusion should be sought through continued optimization of BT strategies. Future directions include earlier utilization and planning for CAR-T to minimize the need for aggressive BT. The role of T-cell redirecting bispecific antibodies (bsAb) as a BT modality remains unclear as well. For triple-class refractory MM, one could consider talquetamab (a GPRC5d targeting bsAb) as BT prior to a BCMA-targeting CAR-T. Continued efforts such as these are warranted to transform CAR-T into a curative modality!

As optimization of outcomes for CAR-T therapy continues with novel/dual targets, rapid manufacturing, and maintenance approaches; one key avenue for improving outcomes is focusing on the need and type of BT. As research continues with constructs such as allogeneic CAR-T, academic-based, and rapidly manufactured autologous CAR-Ts; we may reach a point where BT is not a requisite. By reducing “vein-to-vein” time, many patients may not need/have time for any intervening therapy. However, BT may still be required even in those cases to debulk disease and optimize outcomes. Efforts to minimize/eliminate BT are additionally valuable as BT itself (especially more aggressive BT) can have significant morbidity in deference to cytopenias and infectious risk.

This study elucidates not only the importance of disease biology but also the role of management prior to apheresis. Having aggressive disease immediately prior to CAR-T continues to have a negative impact on the outcome even if BT is administered. If CAR-T is going to be an nth-line therapy, start preparing at line n-1. This includes referral to a CAR-T center or internal preparation for CAR-T during the previous line, prior to progression. Initiation of the logistical processes of CAR-T early in the cadence of progressive disease and not later. This may help to provide a more optimized patient who may not require extensive (or perhaps any) BT.
